# Urethral Caruncle, Their Treatment

**Published:** 1891-06

**Authors:** J. W. Hamilton

**Affiliations:** Lampasas, Texas


For Daniel's Texas Medical Journal.

### URETHRAL CARUNCLE AND THEIR TREATMENT.

BY J. W. HAMILTON, M. D., LAMPASAS, TEXAS.

[Read at the Austin District Medical Society.]

OF ALL the minor troubles that fall to the care of the practicioner, probably there is not one that worries the patient and taxes the ingenuity of the physician more than caruncle of the female urethra.

Near the edge of the meatus, and sometimes along the urethral canal, little vascular, or hemorroidal tumors develop themselves, their color varying from scarlet to dark bluish red. They may consist of a single tumor, or there may be a cluster of small ones, and sometimes a conglomeration of mucous membrane cysts and vascular tumors with new formations, together extending along the urethral canal. According to Castle, Perry, Reid, Wedl, and others, they consist of hypertrophied papilla, which as they enlarge are accompanied by excessive growth of areola tissue. They are extremely vascular, and are richly supplied with nervous filaments. It is thought that chronic urethritis, with proliferation of underlying connective tissue, is the most important etiological factor.

From all the accessible literature on the subject, I find that the treatment heretofore has been far from satisfactory, and consists in either caustic application or removal by some of the various devices at our command. Dr. Thomas says, "The treatment of this condition is most unsatisfactory. I have met with a number of cases of marked character, and in not one was complete relief given by treatment," and closes his remarks by saying, "my observation of the results of caustics and the knife is not such as to inspire me with confidence in them."

So strong an advocate of nitric acid declares that caustics will not cure, but later advises the thermo cautery.

Dr. Bell recommends removal by knife or scissors. The late Dr. Beatty treated them by passing a ligature of fine wire around them with Wilde's snare for aural polypi, and twisting them off.

All authors that I have consulted speak of the tendency to recur when treated by any of the above mentioned methods.

Here I wish to report two cases treated successfully by myself. During the fall of 1888, I was consulted by Mrs. A., who informed me that she had been a constant sufferer for several years, with bladder trouble, which was growing worse. She urinated about every hour, and suffered such agonizing pains that the nervous system had become impaired to an alarming extent. She had no appetite, was unable to sleep, was somewhat anæmic, and extremely hysterical. She stated that she had been treated for "womb disease" a number of times, and had taken large quantities of nauseous medicines for "irritable bladder." Upon examination, I found, just peeping from the meatus, a scarlet red body, which proved to be a vascular tumor of the urethra with a broad, flat base, about the size of an acorn. I treated it with caustics a few times, and succeeded in making matters worse, if possible. I then proposed to remove with scissors. But my patient, like the woman in New Testament times, had "suffered many things of many physicians," and growing no better, would not consent to the operation. Then the idea occurred to me that if hemorrhoidal tumors of the rectum could be cured by injecting carbolic acid, why not address the same treatment to urethral hemorrhoids? Immediately acting upon the idea, I brushed the parts with an eight per cent. solution of cocaine, and injected the tumor with glycerole of carbolic acid. The result was splendid. My patient improved from that hour, and was well in two weeks. Anæmia and hysterical symptoms yielded to tonic treatment.

CASE 2. In July, 1890, I was called to see Mrs. B., with history somewhat similar to the preceding case, and general condition much worse. The patient was very anæmic and hysterical. She had a cough, and suspicion of consumption existed.

She said she had suffered from painful micturation at times for nine years, but for the last six months her suffering had been so great as to embitter her whole life, and if she could not obtain relief she did not wish to live. Upon examination, I found a cluster of little vascular tumors extending along the urethral canal; they were exquisitely tender, and bled from the slightest touch. Dr. Lincecum was in the case with me, and owing to difficulty in reaching the tumors with scissors, and the hemorrhage likely to follow excision, I advised injection, which Dr. L. agreed to. Accordingly we brushed the parts with a strong solution of cocaine, and injected the tumors in reach with Shuford's mixture. The patient experienced great relief from this treatment. Three weeks later I returned, and dilated the urethral canal thoroughly with Allen's surgical pump and rubber bag. I then injected the tumors lying further along the canal. This operation was not followed by any pain. A slight incontinence of urine followed as the result of the dilation, but passed off in a few days. The urethral trouble was completely cured. In carbolic acid injections we recognize an old principle, and new only in the point of application.

Fortunately, urethral caruncle are not very common; but feeling that any practitioner will hail with delight any simple and efficient means of producing a radical cure of such a troublesome condition as I have described, I have been led to report these cases and results, and I hope that should any of the gentlemen present meet with an opportunity, they will give the treatment a trial and let us hear from them.

